# Supplementary material for: Hot spot prediction in protein-protein interactions by an ensemble system
Source: BMC Syst Biol. 2018 Dec 31;12(Suppl 9):132. doi: 10.1186/s12918-018-0665-8 (PMC6311905; doi:10.1186/s12918-018-0665-8)
Supplement: Supplementary file 2 — All relevant MATLAB code. A simple Matlab implement of our predictor. (ZIP 19,742 kb) [file 12918_2018_665_MOESM2_ESM.zip › Additional file 2/Read ASA/ASA website/SKEMPI/1A22A.htm]

 NetSurfP - Protein Surface Accessibility and Secondary Structure Predictions

  

|  |  |  |
| --- | --- | --- |
|  |  | NetSurfP - Protein Surface Accessibility and Secondary Structure Predictions  Technical University of Denmark |

  


---

```
# For publication of results, please cite:
# A generic method for assignment of reliability scores applied to solvent accessibility predictions.
# Bent Petersen, Thomas Nordahl Petersen, Pernille Andersen, Morten Nielsen and Claus Lundegaard
# BMC Structural Biology 2009, 9:51 doi:10.1186/1472-6807-9-51
#
# Column 1: Class assignment - B for buried or E for Exposed - Threshold: 25% exposure, but not based on RSA
# Column 2: Amino acid
# Column 3: Sequence name
# Column 4: Amino acid number
# Column 5: Relative Surface Accessibility - RSA
# Column 6: Absolute Surface Accessibility
# Column 7: Z-fit score for RSA prediction
# Column 8: Probability for Alpha-Helix
# Column 9: Probability for Beta-strand
# Column 10: Probability for Coil
E F  Sequence               1    0.730 146.431  -1.619   0.003   0.003   0.994
E P  Sequence               2    0.593  84.203  -0.991   0.018   0.019   0.964
E T  Sequence               3    0.609  84.441  -0.104   0.058   0.017   0.925
E I  Sequence               4    0.338  62.567  -0.618   0.058   0.017   0.925
E P  Sequence               5    0.373  52.957   0.388   0.115   0.016   0.868
B L  Sequence               6    0.122  22.283  -0.392   0.782   0.003   0.216
E S  Sequence               7    0.522  61.155   1.086   0.858   0.002   0.139
E R  Sequence               8    0.489 112.073   0.713   0.923   0.002   0.076
B L  Sequence               9    0.059  10.748   0.897   0.970   0.001   0.030
B F  Sequence              10    0.075  15.133   0.512   0.970   0.001   0.030
E D  Sequence              11    0.426  61.343   0.326   0.970   0.001   0.030
B N  Sequence              12    0.282  41.358   0.911   0.970   0.001   0.030
B A  Sequence              13    0.025   2.799   0.308   0.970   0.001   0.030
B M  Sequence              14    0.098  19.710  -0.520   0.970   0.001   0.030
E L  Sequence              15    0.328  60.112   0.326   0.970   0.001   0.030
B R  Sequence              16    0.123  28.121   0.529   0.970   0.001   0.030
B A  Sequence              17    0.024   2.689  -0.226   0.923   0.002   0.076
E H  Sequence              18    0.349  63.556   0.314   0.970   0.001   0.030
E R  Sequence              19    0.292  66.822   0.834   0.970   0.001   0.030
B L  Sequence              20    0.031   5.603   0.467   0.970   0.001   0.030
B H  Sequence              21    0.206  37.508   0.545   0.970   0.001   0.030
E Q  Sequence              22    0.454  81.084   1.735   0.970   0.001   0.030
B L  Sequence              23    0.109  19.885   1.262   0.970   0.001   0.030
B A  Sequence              24    0.027   2.953   0.046   0.970   0.001   0.030
E F  Sequence              25    0.359  72.071   0.921   0.988   0.000   0.012
E D  Sequence              26    0.424  61.142   1.248   0.988   0.000   0.012
B T  Sequence              27    0.038   5.257   0.439   0.970   0.001   0.030
B Y  Sequence              28    0.119  25.345   0.894   0.970   0.001   0.030
E Q  Sequence              29    0.442  79.030   1.885   0.970   0.001   0.030
E E  Sequence              30    0.270  47.169   0.960   0.970   0.001   0.030
B F  Sequence              31    0.036   7.125   0.214   0.970   0.001   0.030
B E  Sequence              32    0.162  28.284   0.194   0.923   0.002   0.076
E E  Sequence              33    0.616 107.650   0.629   0.858   0.002   0.139
E A  Sequence              34    0.459  50.538   0.598   0.694   0.003   0.303
B Y  Sequence              35    0.148  31.628   0.615   0.502   0.002   0.495
B I  Sequence              36    0.073  13.524  -1.401   0.181   0.016   0.803
E P  Sequence              37    0.366  51.964  -0.362   0.115   0.016   0.868
E K  Sequence              38    0.549 112.888  -1.525   0.115   0.016   0.868
E E  Sequence              39    0.563  98.426  -1.306   0.115   0.016   0.868
B Q  Sequence              40    0.275  49.204  -1.220   0.113   0.043   0.844
B K  Sequence              41    0.308  63.356  -1.046   0.118   0.150   0.732
B Y  Sequence              42    0.307  65.691  -1.481   0.125   0.227   0.648
B S  Sequence              43    0.278  32.605  -1.414   0.125   0.227   0.648
E F  Sequence              44    0.335  67.255  -1.412   0.199   0.152   0.649
B L  Sequence              45    0.296  54.289  -0.966   0.216   0.235   0.548
E Q  Sequence              46    0.429  76.566  -0.535   0.216   0.235   0.548
E N  Sequence              47    0.310  45.399  -1.090   0.113   0.087   0.800
E P  Sequence              48    0.457  64.877  -1.247   0.113   0.087   0.800
E Q  Sequence              49    0.368  65.671  -1.662   0.053   0.043   0.903
B T  Sequence              50    0.294  40.750  -1.518   0.018   0.047   0.935
E S  Sequence              51    0.381  44.712  -2.286   0.018   0.047   0.935
B L  Sequence              52    0.273  50.023  -0.628   0.018   0.088   0.893
B C  Sequence              53    0.041   5.784  -1.833   0.018   0.088   0.893
B F  Sequence              54    0.178  35.765   0.160   0.018   0.088   0.893
B S  Sequence              55    0.262  30.730  -1.683   0.018   0.088   0.893
E E  Sequence              56    0.494  86.354  -1.586   0.019   0.141   0.840
E S  Sequence              57    0.316  37.047  -1.572   0.019   0.141   0.840
B I  Sequence              58    0.153  28.361   0.078   0.018   0.088   0.893
E P  Sequence              59    0.584  82.827  -0.399   0.018   0.047   0.935
B T  Sequence              60    0.240  33.233  -0.125   0.005   0.015   0.979
B P  Sequence              61    0.265  37.575  -1.065   0.018   0.019   0.964
E S  Sequence              62    0.695  81.477  -0.572   0.018   0.019   0.964
E N  Sequence              63    0.581  85.088  -0.428   0.109   0.005   0.886
E R  Sequence              64    0.424  97.119  -0.147   0.430   0.016   0.555
E E  Sequence              65    0.672 117.381  -0.194   0.522   0.016   0.462
E E  Sequence              66    0.490  85.655   0.734   0.455   0.046   0.498
B T  Sequence              67    0.157  21.762  -0.708   0.502   0.102   0.396
E Q  Sequence              68    0.325  57.956   0.236   0.428   0.171   0.402
E Q  Sequence              69    0.643 114.804   0.031   0.199   0.152   0.649
B K  Sequence              70    0.194  39.926   0.739   0.113   0.087   0.800
E S  Sequence              71    0.534  62.550  -0.221   0.018   0.047   0.935
E N  Sequence              72    0.384  56.262  -0.629   0.694   0.003   0.303
E L  Sequence              73    0.472  86.350  -0.424   0.923   0.002   0.076
E E  Sequence              74    0.373  65.181   0.146   0.923   0.002   0.076
B L  Sequence              75    0.047   8.606   0.261   0.970   0.001   0.030
B L  Sequence              76    0.062  11.279   0.181   0.970   0.001   0.030
E R  Sequence              77    0.348  79.738   0.613   0.970   0.001   0.030
B I  Sequence              78    0.110  20.257  -0.216   0.970   0.001   0.030
B S  Sequence              79    0.040   4.747  -1.057   0.970   0.001   0.030
B L  Sequence              80    0.122  22.338  -0.739   0.970   0.001   0.030
B L  Sequence              81    0.231  42.351  -0.952   0.970   0.001   0.030
B L  Sequence              82    0.079  14.410  -0.160   0.970   0.001   0.030
B I  Sequence              83    0.074  13.709  -0.967   0.923   0.002   0.076
E Q  Sequence              84    0.303  54.152  -0.607   0.858   0.002   0.139
B S  Sequence              85    0.047   5.567  -1.779   0.600   0.003   0.397
B W  Sequence              86    0.066  15.897  -1.207   0.502   0.002   0.495
E L  Sequence              87    0.271  49.620  -0.489   0.600   0.003   0.397
E E  Sequence              88    0.374  65.285  -0.017   0.502   0.002   0.495
B P  Sequence              89    0.049   6.981  -0.795   0.694   0.003   0.303
B V  Sequence              90    0.072  11.097  -1.386   0.858   0.002   0.139
E Q  Sequence              91    0.432  77.084   0.919   0.923   0.002   0.076
B F  Sequence              92    0.243  48.730   0.452   0.923   0.002   0.076
B L  Sequence              93    0.028   5.090   0.542   0.923   0.002   0.076
E R  Sequence              94    0.277  63.410   0.226   0.858   0.002   0.139
E S  Sequence              95    0.539  63.124   0.090   0.782   0.003   0.216
B V  Sequence              96    0.283  43.543  -1.000   0.600   0.003   0.397
B F  Sequence              97    0.162  32.554  -0.949   0.321   0.003   0.675
E A  Sequence              98    0.483  53.205  -1.363   0.109   0.005   0.886
E N  Sequence              99    0.484  70.872  -1.624   0.058   0.017   0.925
E S  Sequence             100    0.420  49.165  -1.618   0.058   0.017   0.925
E L  Sequence             101    0.486  88.913  -2.122   0.115   0.016   0.868
E V  Sequence             102    0.435  66.875  -2.060   0.115   0.016   0.868
B Y  Sequence             103    0.226  48.253  -1.039   0.184   0.043   0.773
B G  Sequence             104    0.287  22.571  -1.074   0.268   0.043   0.689
B A  Sequence             105    0.314  34.636  -1.972   0.268   0.043   0.689
E S  Sequence             106    0.356  41.665  -0.394   0.455   0.046   0.498
B D  Sequence             107    0.200  28.806  -0.778   0.802   0.014   0.185
E S  Sequence             108    0.502  58.811   0.988   0.938   0.007   0.055
E N  Sequence             109    0.350  51.181   0.452   0.923   0.002   0.076
B V  Sequence             110    0.040   6.225  -0.026   0.970   0.001   0.030
E Y  Sequence             111    0.281  60.007   0.767   0.970   0.001   0.030
E D  Sequence             112    0.579  83.362   1.618   0.970   0.001   0.030
B L  Sequence             113    0.184  33.727   0.714   0.970   0.001   0.030
B L  Sequence             114    0.035   6.427  -0.276   0.970   0.001   0.030
E K  Sequence             115    0.509 104.722   1.753   0.970   0.001   0.030
E D  Sequence             116    0.404  58.274   1.842   0.970   0.001   0.030
B L  Sequence             117    0.026   4.742   0.827   0.988   0.000   0.012
E E  Sequence             118    0.275  48.077   0.592   0.988   0.000   0.012
E E  Sequence             119    0.566  98.810   1.235   0.988   0.000   0.012
B R  Sequence             120    0.162  36.983   0.011   0.988   0.000   0.012
B I  Sequence             121    0.028   5.254   0.466   0.988   0.000   0.012
E Q  Sequence             122    0.410  73.262   1.442   0.988   0.000   0.012
E T  Sequence             123    0.294  40.764   1.180   0.988   0.000   0.012
B L  Sequence             124    0.057  10.510   0.334   0.970   0.001   0.030
B M  Sequence             125    0.058  11.606  -0.160   0.975   0.003   0.022
E G  Sequence             126    0.495  38.996   0.607   0.938   0.007   0.055
E R  Sequence             127    0.464 106.279   0.350   0.879   0.010   0.111
B L  Sequence             128    0.127  23.345  -0.727   0.717   0.014   0.269
E E  Sequence             129    0.342  59.747  -0.501   0.339   0.016   0.645
E G  Sequence             130    0.483  37.981  -1.702   0.257   0.016   0.727
E Q  Sequence             131    0.458  81.781  -1.448   0.115   0.016   0.868
E I  Sequence             132    0.306  56.628  -1.575   0.113   0.043   0.844
E F  Sequence             133    0.387  77.691  -0.668   0.113   0.087   0.800
E K  Sequence             134    0.611 125.642  -1.162   0.053   0.043   0.903
E Q  Sequence             135    0.492  87.782  -1.333   0.053   0.043   0.903
E T  Sequence             136    0.503  69.780  -1.536   0.018   0.047   0.935
B Y  Sequence             137    0.196  41.949   0.042   0.018   0.047   0.935
E S  Sequence             138    0.311  36.449  -1.316   0.018   0.019   0.964
E K  Sequence             139    0.551 113.402  -1.843   0.018   0.019   0.964
E F  Sequence             140    0.341  68.399  -1.616   0.058   0.017   0.925
E D  Sequence             141    0.574  82.713  -1.378   0.181   0.016   0.803
E T  Sequence             142    0.431  59.794  -2.024   0.522   0.016   0.462
E D  Sequence             143    0.455  65.594  -0.888   0.694   0.003   0.303
E A  Sequence             144    0.444  48.951   0.208   0.694   0.003   0.303
B L  Sequence             145    0.138  25.213  -0.135   0.802   0.014   0.185
B L  Sequence             146    0.221  40.428  -0.041   0.802   0.014   0.185
E K  Sequence             147    0.481  98.983   0.236   0.717   0.014   0.269
E N  Sequence             148    0.284  41.592  -0.589   0.622   0.015   0.363
B Y  Sequence             149    0.211  45.112   0.728   0.430   0.016   0.555
E G  Sequence             150    0.365  28.694  -0.781   0.339   0.016   0.645
B L  Sequence             151    0.089  16.241   0.451   0.455   0.046   0.498
B L  Sequence             152    0.100  18.292  -0.192   0.605   0.105   0.290
B Y  Sequence             153    0.295  62.977  -0.065   0.701   0.107   0.192
B C  Sequence             154    0.047   6.585  -0.779   0.605   0.105   0.290
B F  Sequence             155    0.075  15.093   0.079   0.605   0.105   0.290
E R  Sequence             156    0.349  79.944   0.681   0.386   0.097   0.517
E K  Sequence             157    0.426  87.587   0.822   0.268   0.043   0.689
B D  Sequence             158    0.165  23.805  -0.681   0.339   0.016   0.645
B M  Sequence             159    0.092  18.429  -0.646   0.694   0.003   0.303
E D  Sequence             160    0.482  69.456   0.066   0.858   0.002   0.139
E K  Sequence             161    0.269  55.251   1.852   0.938   0.007   0.055
B V  Sequence             162    0.022   3.351   1.193   0.970   0.001   0.030
E E  Sequence             163    0.314  54.926   0.432   0.970   0.001   0.030
B T  Sequence             164    0.199  27.574   0.600   0.970   0.001   0.030
B F  Sequence             165    0.076  15.313   0.814   0.975   0.003   0.022
B L  Sequence             166    0.052   9.613   1.287   0.938   0.007   0.055
E R  Sequence             167    0.404  92.493   1.583   0.938   0.007   0.055
B I  Sequence             168    0.086  15.855   0.102   0.879   0.010   0.111
B V  Sequence             169    0.059   9.099   0.404   0.779   0.100   0.120
E Q  Sequence             170    0.317  56.580   0.931   0.578   0.229   0.194
B C  Sequence             171    0.061   8.607  -0.120   0.252   0.423   0.325
E R  Sequence             172    0.308  70.532   0.749   0.169   0.612   0.219
B S  Sequence             173    0.233  27.261  -0.498   0.079   0.592   0.329
B V  Sequence             174    0.187  28.757   0.296   0.069   0.386   0.545
E E  Sequence             175    0.594 103.824   0.055   0.053   0.043   0.903
E G  Sequence             176    0.616  48.456  -0.178   0.058   0.017   0.925
E S  Sequence             177    0.490  57.428  -0.614   0.018   0.047   0.935
B C  Sequence             178    0.128  17.929  -1.817   0.019   0.141   0.840
E G  Sequence             179    0.493  38.783  -0.021   0.019   0.141   0.840
E F  Sequence             180    0.506 101.594  -1.060   0.003   0.003   0.994

Explain the output.  Go back.
```

---
